# Computer Vision from Tea Cultivation to Quality Evaluation

**DOI:** 10.3390/foods15162864

**Published:** 2026-08-17

**Authors:** Zunren Chen, Jinfeng Wang, Yilan Sun, Jie Pang, Wei Xin, Qinhua Zhang, Junling Zhou

**Affiliations:** 1 Library of Fujian University of Traditional Chinese Medicine, Fuzhou 350122, China; c2021010@fjtcm.edu.cn; 2 School of Food Science, Fujian Agriculture and Forestry University, Fuzhou 350002, China; 18674272913@163.com (J.W.); yilansun@126.com (Y.S.); pang3721941@163.com (J.P.); 3 College of Ecology and Resources Engineering, Wuyi University, Wuyishan 354300, China; xinwei@wuyiu.edu.cn; 4 Fujian Key Laboratory of Big Data Application and Intellectualization for Tea Industry, Wuyi University, Nanping 529000, China; 5 Jinshan College, Fujian Agriculture and Forestry University, Fuzhou 350002, China; 6 Haixia Institute of Science and Technology, Fujian Agriculture and Forestry University, Fuzhou 350002, China

**Keywords:** artificial intelligence, computer vision, deep learning, smart agriculture, tea industry

## Abstract

Existing reviews on AI in tea production are either agriculture-generic or limited to isolated tasks. This review thoroughly compares vision technologies (RGB, hyperspectral, near-infrared, thermal, Light Detection and Ranging (LiDAR), Unmanned Aerial Vehicle (UAV)) and establishes a task-oriented algorithm selection framework for the tea industry. For small-sample or near-linear problems, traditional machine learning (ML) (support vector machine (SVM); partial least squares regression (PLSR)) remains effective. For unstructured field tasks, deep learning achieves superior performance: pest detection accuracy exceeds 97%, tea bud detection reaches 96.8% with RGB images, and hyperspectral imaging predicts nitrogen content with R^2^ > 0.90 and tea polyphenols with R^2^ up to 0.925. Algorithm choice further differentiates by task granularity: lightweight convolutional neural networks (CNNs) balance speed and accuracy for edge deployment at 16 fps; You Only Look Once (YOLO) series detectors enable real-time localization on mobile platforms at 93.1% accuracy, 24 ms per target. No single algorithm dominates all tea tasks; selection is a trade-off among accuracy, speed, data availability, and computational constraints. These findings outline a structured analysis of the challenges and pathways for transitioning computer vision (CV) from laboratory research toward field-deployable tools.

## 1. Introduction

Tea, an ancient beverage originating in the East, is now the second most consumed drink globally after water [1]. It constitutes a major global industry at the intersection of culture, health, and economics. According to the Food and Agriculture Organization (FAO), global tea production exceeded 6.5 million tons in 2022, with a trade value of approximately $17 billion USD (FAO, 2022), serving as an economic pillar in many rural and developing regions [2]. On one hand, consumer demand is shifting towards higher, more standardized levels of quality, safety, and traceability [3]. On the other hand, structural issues within the industry are intensifying. Major tea-producing regions worldwide are experiencing an aging rural population and a migration of young labor to urban areas, making it increasingly difficult to secure sufficient labor for harvest and other tasks, while costs continue to rise [4]. Furthermore, traditional production is heavily reliant on empirical judgment from fertilization and pest control to harvesting standards and processing, hindering standardization, resulting in inconsistent product quality and difficulties in achieving scalable, high-grade output [5]. This reliance on subjective, experience-based methods represents a fundamental bottleneck to modernization and sustainable development [6].

The advent of new-generation AI, particularly deep learning, has driven transformative breakthroughs across various fields, including agriculture [7]. Within this, AI-powered CV stands out as a key to enabling technology. Capable of automated recognition, measurement, and analysis from image and video data, CV can match or surpass human visual perception. This offers a powerful toolkit to address the traditional constraints of the tea industry [8]. Applications range from macro-scale monitoring of plantation growth and nutrient status using drone-mounted multispectral cameras [9,10] to real-time pest and disease identification via CNNs and precise detection of harvestable tea buds in complex natural environments using object detection algorithms [11]. Moreover, technologies like hyperspectral imaging (HSI) enable non-destructive, real-time monitoring of biochemical constituents during critical processing stages such as withering and fermentation, facilitating digital and precise process control [12].

To provide a comprehensive yet focused overview, this review adopts a pragmatic definition of CV that extends beyond conventional image-based techniques. Specifically, we include not only RGB, HSI, and thermal imaging (TIC) but also near-infrared spectroscopy (NIRS), LiDAR, and UAV [13]. These technologies can be categorized into imaging modalities (RGB, HSI, TIC), spectroscopic techniques (NIRS), 3D spatial sensors (LiDAR), and aerial platforms (UAV). NIRS is considered because, in tea processing, it is often integrated with imaging systems for real-time quality prediction [14]; LiDAR is included for its ability to provide three-dimensional spatial information that complements 2D images for bud localization and canopy analysis [15], and UAV is treated as a platform that carries vision sensors, enabling large-scale, multi-temporal monitoring [11]. The selection of these technologies was guided by three practical criteria-scalability (RGB cameras and UAVs can cover large areas), robustness under field conditions (LiDAR’s insensitivity to lighting variation), and feasibility for real-world deployment (NIRS portability for inline processing). This broad yet practical scope reflects the multimodal sensing strategies commonly employed in current tea industry research. Given this focus on practical application, we prioritize studies that report quantitative performance metrics, whether conducted in the laboratory, pilot line, or field.

The literature covered in this review was primarily identified through searches in Web of Science, Scopus, and Google Scholar using keywords including “computer vision,” “deep learning,” “tea industry,” “precision agriculture,” “hyperspectral imaging,” “NIRS,” “LiDAR,” and “UAV-based remote sensing.” The search covered publications from 2018 to 2025, with emphasis on studies reporting quantitative performance metrics. References were selected based on their relevance to the tea production chain and their contribution to the comparative analysis of vision technologies and algorithms.

Recent reviews have summarized AI applications in tea management, harvesting, and processing, but a quantitative, full-chain comparison of vision technologies with a dedicated algorithm selection framework has been less explored. Unlike existing reviews that focus on isolated tasks or general agriculture, this review provides a quantitative comparison of vision technologies alongside a task-oriented algorithm selection framework across the entire tea production chain. It offers critical comparative analyses of technological approaches, identifies practical constraints, and examines translational challenges from lab to field, aiming to bridge the gap between research and industrial adoption.

## 2. Key Enabling Technologies and Data Acquisition Systems in the Tea Industry

### 2.1. AI-Powered CV Technology: Core Concepts for Tea Applications

**Figure 1:**
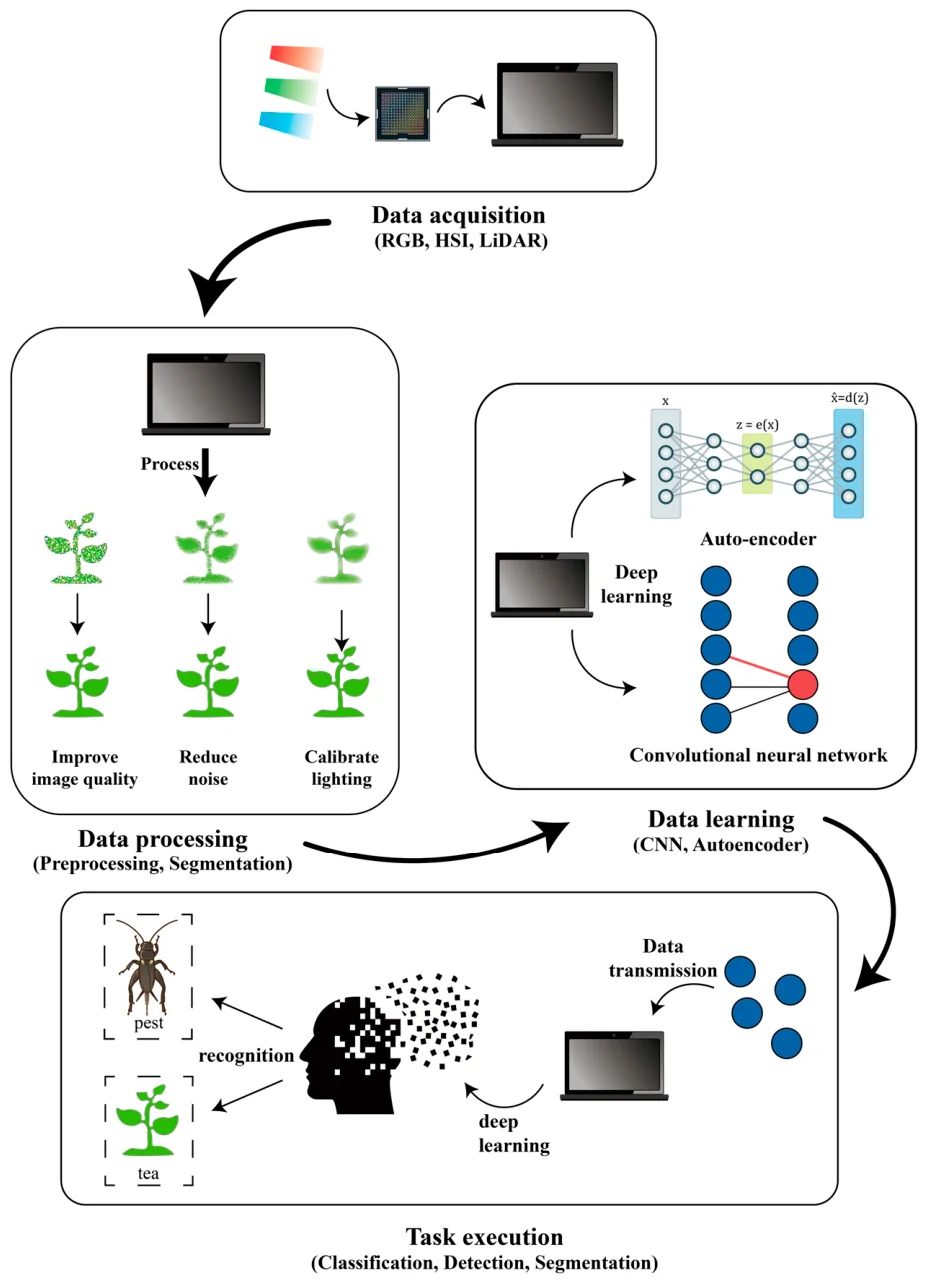
Typical workflow of CV systems for tea applications. The red circles and lines indicate the propagation paths selected by the proposed strategy for the convolutional neural network.

AI-powered CV integrates CV and deep learning to automatically extract information from images. Its pipeline typically includes data acquisition using RGB, HSI, TIC, or LiDAR sensors, data processing (preprocessing, segmentation), feature learning (via CNNs, autoencoders), and task execution (classification, detection, segmentation). In the tea industry, deep learning, especially CNNs, has become dominant for handling complex, unstructured scenes such as pest detection and bud recognition, while traditional ML (SVM, PLSR) remains useful for small-sample or near-linear problems. The operational framework of a CV system typically mirrors human visual processing, encompassing four key stages: data acquisition, data processing, data learning, and task execution (see Figure 1).

### 2.2. Multidimensional Visual Perception Technology

The efficacy of CV systems in the tea industry is fundamentally dependent on the underlying perception technologies for information acquisition. The detailed comparison of these technologies, including their core benefits, key applications, performance examples, validation settings, cost, maturity levels, and spatial resolution, is presented in Table 1. Unlike human vision, which is confined to the visible spectrum, modern perception technologies construct a multidimensional visual perception system that vastly exceeds human sensory capabilities by expanding the spectral range and integrating spatial, temporal, and chemical dimensions. This system provides holistic insights from macro to micro scales and from external morphology to internal chemistry, enabling intelligent monitoring and decision-making across the entire tea production chain, from cultivation to processing.

In terms of spatial resolution (SR) and acquisition speed, the six technology categories differ substantially. RGB cameras typically achieve 0.1–1 mm/pixel at 30–60 fps. HSI offers similar spatial resolution but much slower acquisition (0.1–10 fps) due to scanning. NIRS provides no spatial image (point/line scan) but is fast (10–100 spectra/s). TIC gives lower spatial resolution (1–5 mm/pixel) at 1–30 fps. LiDAR generates 3D point clouds with cm-to-dm resolution at 10–100 k points/s. UAV-based remote sensing covers large areas at 0.5–5 cm/pixel per flight (10–30 min per 50 ha). A rough cost ranking (low to high) is: RGB < NIRS < TIC ≈ UAV < HSI ≈ LiDAR. At the lower end, RGB cameras and consumer-grade NIRS devices are relatively affordable and widely available. TIC and UAV systems fall into the medium range, though costs vary considerably depending on sensor resolution and payload capacity. HSI and LiDAR remain the most expensive, due to their high-precision optics, complex calibration procedures, and substantial data storage and computational demands. To facilitate the comparison of technological maturity, each technology is assessed using the Technology Readiness Level (TRL), where levels 1–3 indicate basic research and proof-of-concept, 4–6 indicate validation in laboratory or relevant environments, and 7–9 indicate system prototype demonstration and full operational deployment.

#### 2.2.1. RGB Imaging

RGB imaging, which simulates human trichromatic vision, is the most widely adopted visual perception technology due to its low cost and simple deployment. In the tea industry, RGB cameras are primarily used to capture morphological, color, and textural features for tasks such as identifying bud maturity, preliminary screening of pronounced foliar disease lesions, and grading the appearance and color of dry tea. However, RGB imaging is inherently limited to surface reflectance in the visible spectrum. It cannot penetrate surfaces, detect chemical constituents, or effectively identify early-stage, latent physiological changes. Its performance is also highly susceptible to variations in ambient lighting conditions [13]. To mitigate these lighting-induced issues, common preprocessing strategies include histogram equalization, white balance correction, and conversion to HSV or Lab color spaces to separate luminance from chrominance [25]. Adaptive thresholding and illumination normalization can further improve robustness in non-uniform lighting [26].

#### 2.2.2. HSI

HSI, which integrates spectroscopy with imaging, is a core enabling technology for intelligent perception in the tea industry [27]. Unlike an RGB pixel containing only three broad bands, a hyperspectral pixel captures reflectance across hundreds of contiguous narrow bands (typically with spectral resolution < 10 nm), forming a rich data cube with combined spatial and spectral dimensions [28].

This capability stems from the absorption of specific near-infrared photons by vibrational overtones of chemical bonds involving hydrogen (O-H, N-H, C-H). Consequently, subtle changes in key internal chemical components produce distinctive responses in the spectral signature [18]. HSI thus enables non-destructive, spatially resolved chemical mapping—for instance, visualizing moisture distribution during withering, quantifying the dynamics of polyphenol transformation during fermentation, or identifying pre-visual plant stress [19]. In contrast, multispectral imaging (MSI) acquires data at only a few discrete bands, trading spectral resolution for lower cost and faster acquisition. While HSI typically provides higher prediction accuracy for internal chemical constituents (R^2^ > 0.91 for nitrogen and polyphenols [19]), MSI is more suitable for large-scale, real-time field monitoring due to its reduced data volume [29].

The primary challenges associated with HSI are data redundancy and high computational cost [30]. Effective implementation often requires dimensionality reduction via wavelength selection algorithms or leveraging deep learning models like CNNs to automatically extract critical spectral-spatial features [31].

#### 2.2.3. NIRS

NIRS operates on a similar spectroscopic principle to HSI but typically acquires spectral data in a point or line-scan mode, emphasizing rapid, portable, and online analysis [6]. Its high sensitivity to hydrogen-containing groups makes it indispensable for real-time quality control during tea processing [20]. The miniaturization of NIRS with fiber-optic probes facilitates direct integration into production lines for non-destructive, inline monitoring. Key applications include real-time moisture monitoring during fixation and drying for precise thermal control, and objective assessment of fermentation degree by tracking specific spectral features [32].

A current research frontier involves fusing NIR spectral data with RGB image data to leverage both chemical and morphological information for building more robust quality prediction models.

#### 2.2.4. TIC and LiDAR

TIC captures temperature distribution by detecting an object’s emitted infrared radiation. In tea garden management, it can identify canopy temperature anomalies indicative of water stress, providing a direct guide for precision irrigation [33]. However, TIC accuracy is highly sensitive to environmental conditions (ambient temperature, wind, humidity, solar radiation) and leaf emissivity (typically 0.95–0.98 for green vegetation). To obtain reliable data, calibration is essential; common practices include using a reference blackbody, correcting for reflected temperature, applying emissivity adjustment, and measuring under stable weather conditions (midday, clear sky). Without such steps, temperature errors can exceed 2–3 °C, leading to incorrect stress diagnosis [21].

LiDAR, in contrast, provides precise depth information to generate 3D point clouds. This technology is critical for intelligent harvesting, enabling accurate 3D localization of tea buds within complex, overlapping foliage and guiding robotic arms for obstacle avoidance and grasping [34]. Furthermore, LiDAR mounted on UAVs can create detailed 3D digital maps of tea gardens for canopy structure analysis and biomass estimation. However, dense canopy occlusion limits LiDAR performance: laser pulses are often intercepted by upper leaves, resulting in incomplete point clouds of lower buds. Multi-return or full-waveform LiDAR partially mitigates this but still struggles with deep internal structures [22].

#### 2.2.5. UAV

Unmanned Aerial Systems equipped with multispectral, HSI, or RGB cameras form an aerial platform for macro-scale monitoring [24]. They enable rapid, large-area acquisition of high-resolution imagery over difficult terrain. These systems are used to monitor field vigor and uniformity, derive vegetation indices like Leaf Area Index, assess nutrient status, and detect early-stage pest or disease outbreak foci [23]. By analyzing multi-temporal remote sensing data with ML models, it is possible to track growth dynamics and predict yield, providing a scientific basis for farm-level precision management and resource allocation [35].

### 2.3. Data Processing and Feature Engineering

**Figure 2:**
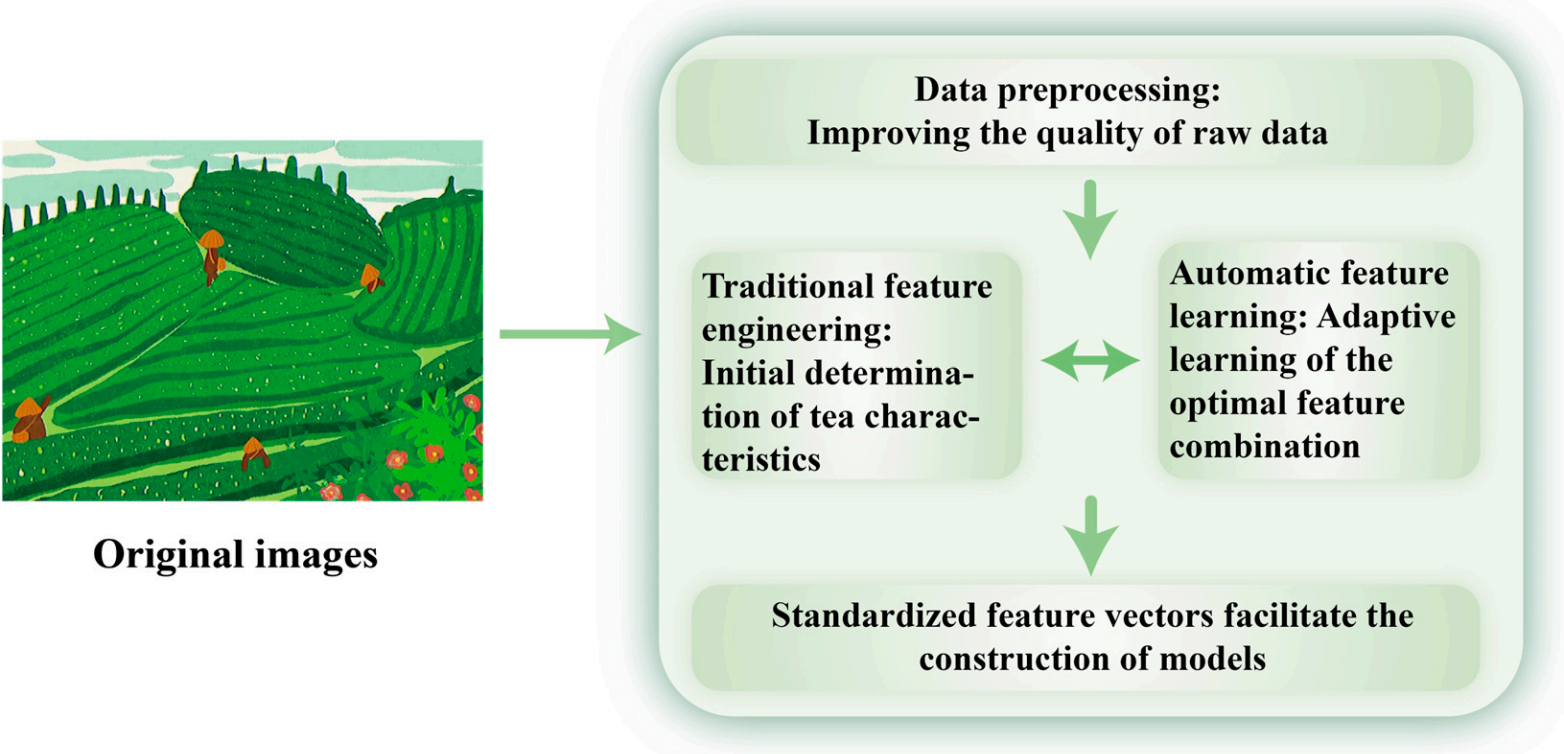
Data processing and feature engineering pipeline for tea image analysis.

Data processing and feature engineering serve as a critical bridge connecting raw visual data to intelligent model decision-making. Their objective is to transform disorganized, raw image information into normalized, discriminative features suitable for effective model learning [36]. Figure 2 illustrates the complete pipeline from raw image data to model-ready features, covering the key steps of preprocessing, segmentation, and feature extraction. Common preprocessing steps include noise filtering, contrast enhancement, geometric correction, and color space conversion to stabilize features under variable lighting [37,38,39]. Segmentation techniques (thresholding, clustering, or lightweight CNNs) isolate targets such as tea buds or disease spots from complex backgrounds [40]. Traditional handcrafted features, including color histograms, color moments, and morphological descriptors, remain valuable for small-sample or interpretability-demanding tasks in tea grading and disease identification [41,42]. The preprocessing workflows are not identical across modalities. RGB preprocessing focuses on illumination correction (histogram equalization, white balance) [43]. Hyperspectral preprocessing requires dark-current removal, noise smoothing, and dimensionality reduction [19]. TIC demands temperature calibration accounting for emissivity, ambient conditions, and sensor distance [21]. Recent studies have also applied histogram equalization to improve thermal-RGB registration for UAV tea monitoring. Thus, while the general pipeline is common, specific operations must be adapted to each modality.

Deep learning, particularly CNNs, has largely automated feature learning by hierarchically extracting edges, textures, and semantic representations directly from raw pixels [44]. This capability provides advantages for tea applications: models can learn optimal feature combinations for distinguishing subtle differences in withering degree or visually similar diseases, often surpassing human-designed features [45,46]. By learning from large datasets, deep models acquire more robust and invariant representations, significantly improving reliability in complex field environments [47]. Architecture enhancements such as attention mechanisms further boost performance for tea image classification, detection, and segmentation tasks.

Within the tea industry, given the unique acquisition environments and target objects, this process is particularly crucial, as it directly determines the performance ceiling and generalization capability of subsequent models [48].

### 2.4. An Analytical Framework for Algorithm Selection

**Figure 3:**
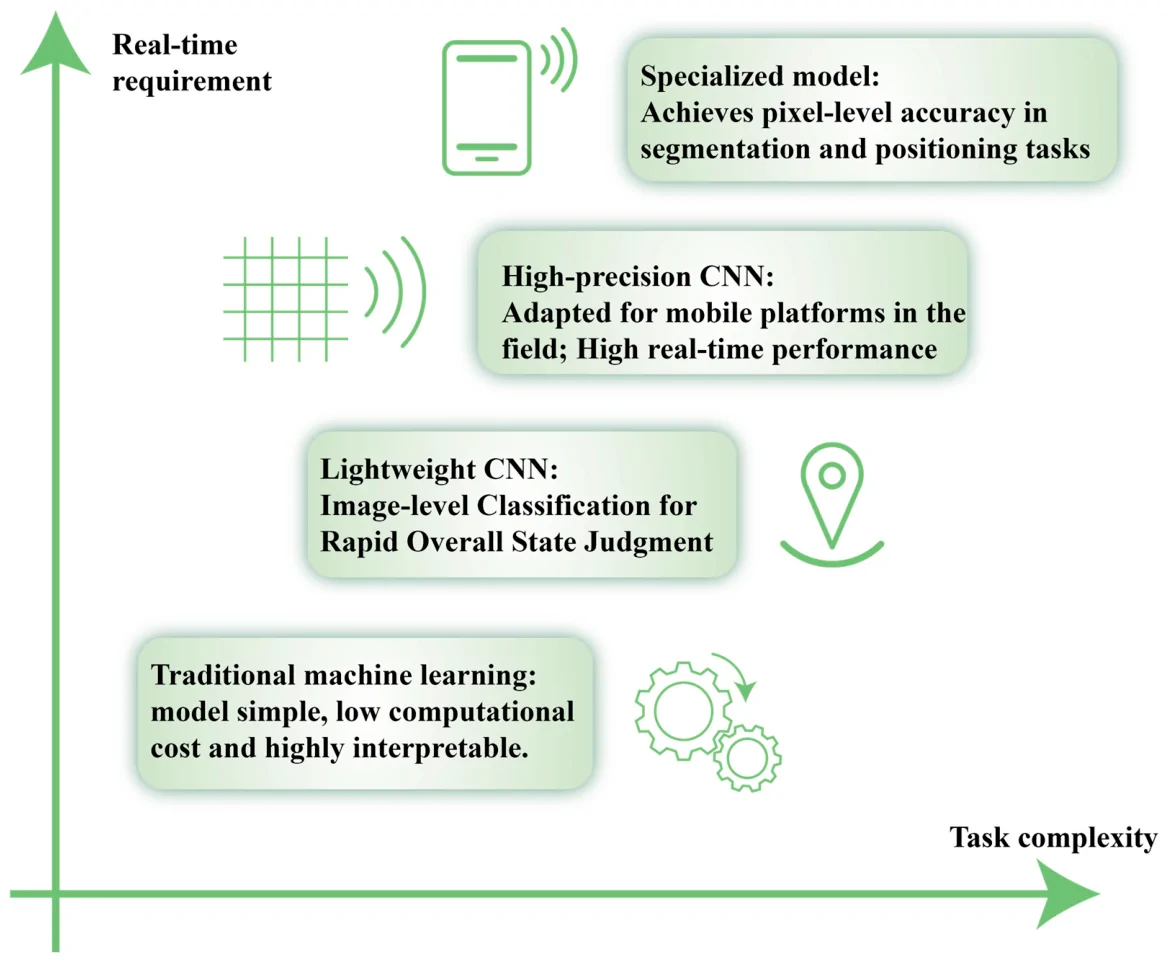
Task-oriented algorithm selection logic.

The selection of CV algorithms in the tea industry is not driven by a single performance metric but constitutes a multi-criteria trade-off decision-making process under specific application constraints. As summarized in Figure 3, the logic for selection is primarily governed by task complexity and real-time requirements, reflecting a clear evolutionary trajectory from traditional methods to deep learning, and further towards specialized, lightweight models [7].

For small-sample or near-linear problems, traditional ML (SVM, PLSR) remains effective due to its low computational cost and interpretability [49]. However, most tea vision tasks, including pest recognition in natural environments, tea bud detection against complex backgrounds, and dynamic quality monitoring during processing, present unstructured challenges that favor deep learning, particularly CNNs, for their ability to learn robust features autonomously [48]. Recent advances in low-cost portable imaging have further broadened accessibility. For instance, a smartphone-based contact imaging method combined with 1-D CNN achieved an R^2^ of 0.82 for tea leaf chlorophyll estimation [50]. A multispectral camera system (coupled with random forest regression) predicted tea quality parameters (polyphenols, amino acids) with R^2^ up to 0.85, offering a cost-effective alternative to expensive hyperspectral instruments [51]. These portable solutions lower the barrier for smallholder tea farmers, although they typically provide lower spectral resolution than laboratory-grade systems.

Task granularity further diversifies the choice. Image-level classification favors lightweight CNNs that balance speed and accuracy on edge devices [52]. For object detection and localization, single-stage detectors like the YOLO series are widely adopted for real-time field deployment on mobile platforms, albeit with higher miss rates for small targets [53]. Pixel-wise fine segmentation relies on Fully Convolutional Networks (FCN) or Mask R-CNN for precision at higher computational cost [54]. Beyond these, U-Net and Deep Lab variants have also been applied to tea bud segmentation, achieving pixel-wise precision at the cost of higher computational load [55]; standard vision transformers (ViT) have not yet been widely applied to tea tasks, but their attention mechanism offers potential for capturing global context in tea canopy or processing images [56].

Despite these advances, a critical gap remains: models trained on one tea cultivar, region, or season often fail to generalize to others. Domain adaptation and few-shot learning have not yet been systematically applied to tea vision tasks, but they hold strong potential to reduce the lab-to-field accuracy drop without extensive re-annotation. We therefore identify them as a key future direction.

Current frontiers also include lightweight model optimization and hybrid CNN-Long Short-Term Memory (LSTM) models for capturing temporal dynamics in tea processing. Table 2 summarizes key comparative dimensions for algorithm selection across typical tea scenarios.

### 2.5. Adaptability and Gaps of Vision Technologies and AI Models Across the Tea Value Chain

The preceding sections have detailed various multidimensional vision technologies and algorithm selection frameworks. However, in practical deployment across the tea production chain, significant differences in adaptability and maturity gaps become evident. This section systematically compares three dimensions: technology-scenario fit, model-task selection, and the critical divide between laboratory research and industrial implementation.

#### 2.5.1. Scenario Adaptability of Multidimensional Vision Technologies

No single vision technology simultaneously meets the requirements of cost, information richness, and deployment ease across the tea production chain. RGB imaging is the workhorse for field applications due to its low cost but fails to sense internal chemistry and is sensitive to lighting [57]. HSI offers detailed chemical mapping but remains largely laboratory-bound because of its high cost and data complexity [58]. NIRS provides rapid, portable monitoring of moisture and fermentation, serving as a pragmatic bridge between lab and production line. LiDAR enables precise 3D localization for robotic harvesting, yet its high cost limits widespread adoption [59]. UAV remote sensing supports large-scale field monitoring but is constrained by regulations, weather, and battery life [60].

**Figure 4:**
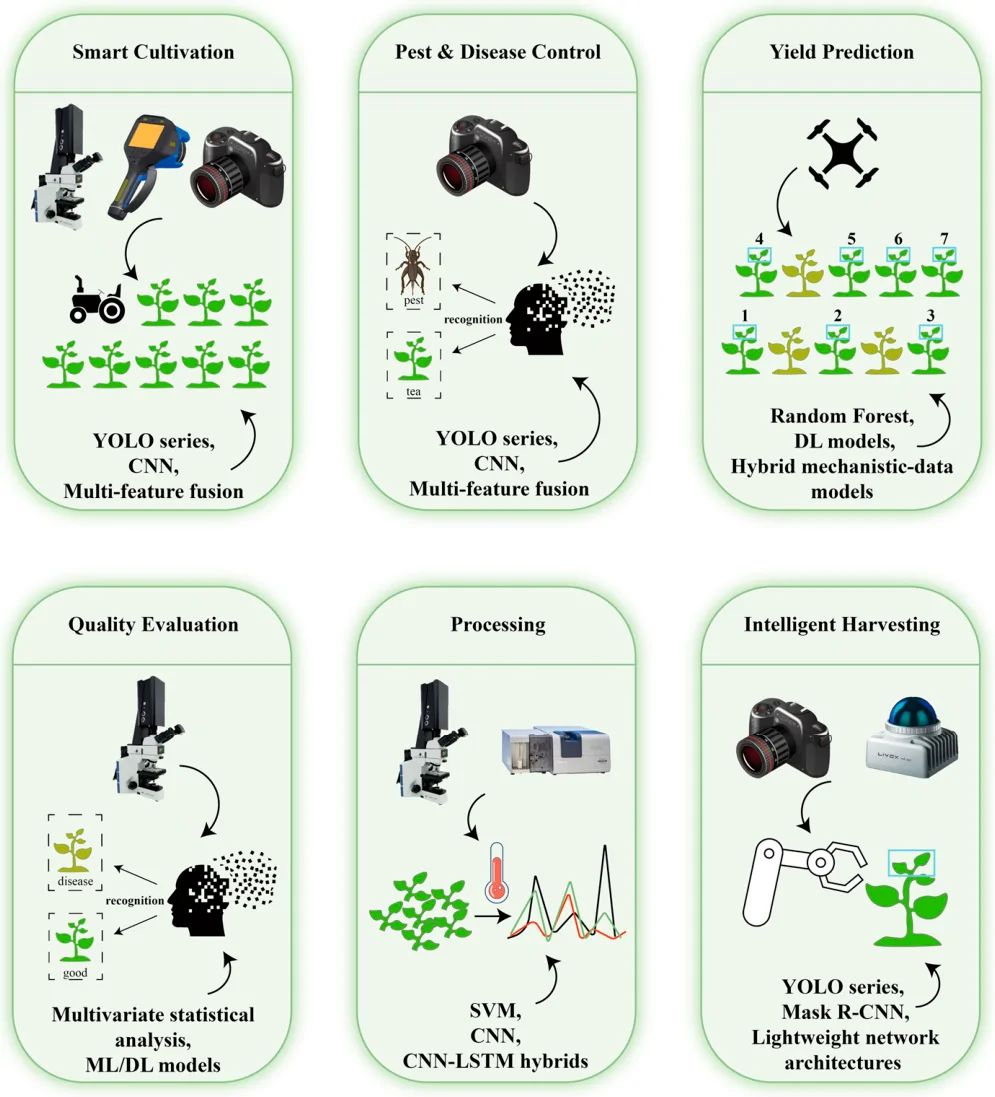
Visual guide to technology selection strategies for different application scenarios along the tea production chain.

In practice, integrating multiple technologies is often necessary; the choice of integration strategy must balance cost, information dimensionality, and deployment feasibility [61]. For instance, combining the spatial detail of RGB with the chemical sensitivity of NIRS or HSI can provide a more complete picture for quality assessment, but the added cost and complexity must be justified by the specific application requirements. Early fusion (combining raw data) is rarely practical for heterogeneous sensors; instead, feature-level fusion (combining extracted features) or decision-level fusion (combining model outputs) are more common in current tea research. Figure 4 visually summarizes the technology selection strategies for different application scenarios along the tea production chain, providing practical guidance for matching sensing technologies to specific task requirements.

#### 2.5.2. Key Environmental Differences Between Laboratory Research and Field Application in Tea Gardens

A persistent gap exists between laboratory research and field application [62]. Table 3 summarizes key environmental differences—lighting variability, background clutter, occlusion, hardware constraints, and environmental stability—that cause models trained under controlled conditions to suffer significant performance drops (typically 15–20 percentage points) when deployed in real tea gardens or factories [63]. While data augmentation, domain adaptation, model compression, and edge computing can mitigate these gaps, most current research remains at the laboratory validation stage. Bridging this divide requires end-to-end adjustments across data acquisition, model design, and evaluation protocols, rather than isolated improvements at any single stage [52,64,65].

## 3. Application of Computer Vision Technology in the Tea Industry

The value of CV in the tea industry lies in its precise synergy of algorithms, equipment, and application scenarios to address enduring bottlenecks across the production chain. It establishes a closed-loop system that integrates perception, analysis, and decision-making, offering systematic solutions. As outlined in Table 4, this section follows the sequence of the production chain to analyze specific challenges, adapted technological setups, core algorithmic principles, and resultant value for different scenarios. To enable meaningful interpretation of the reported results. This section draws on studies from three validation tiers. The first tier is controlled laboratory experiments using high-precision instruments. The second tier comprises pilot-line investigations conducted under simulated or near-production conditions with portable equipment. The third tier includes field studies performed in operational tea gardens or processing facilities under natural conditions. True industrial deployment remains absent, with no system yet operating autonomously in production workflows over extended periods.

### 3.1. Smart Cultivation: Enabling Targeted Fertilization and Growth Tracking

Traditional tea cultivation relies heavily on empirical knowledge, leading to delayed and imprecise responses to variations in climate, soil, and plant growth. CV directly addresses these limitations by enabling real-time, data-driven precision management [77]. Specifically, CV can contribute to more efficient and targeted fertilizer application strategies [77].

UAV or fixed monitoring stations equipped with HSI cameras, thermal infrared sensors, and RGB cameras facilitate large-scale, periodic data acquisition of tea gardens [78]. This overcomes the limitations of manual scouting. Sophisticated algorithms then analyze this imagery. Extending this capability by capturing reflectance across hundreds of narrow bands enables quantitative detection of biochemical constituents [79].

At the laboratory or controlled-environment level, HSI combined with PLSR or CNN models can estimate nitrogen content, chlorophyll, and water stress levels in tea leaves. Yamashita et al. demonstrated that HSI with ML algorithms could predict nitrogen and chlorophyll contents with R^2^ > 0.90 [69]. TIC detects canopy temperature anomalies caused by stomatal closure under water deficit, providing a direct indicator for precision irrigation [80]. When these multi-sensor data are integrated with soil sensors and variable-rate application maps, intelligent machinery can execute site-specific fertilization and irrigation, reducing environmental impact while improving yield and quality.

At the field-deployment level, recent studies have shown that UAV-based MSI with ML can classify tea shoot growth stages and map shoot density distribution across tea plantations, enabling rapid, large-scale assessment of germination patterns and spatial uniformity and achieving 97% F1-score for tea bud detection and 96% for mature leaves [29].

Overall, the fusion of RGB, HSI, and thermal data offers a pathway toward a closed-loop system for precision tea cultivation, confirming that CV can indeed reduce reliance on empirical judgment and enable more efficient, targeted fertilization as well as growth tracking.

It should be noted, however, that most of these studies were conducted on a limited number of cultivars and under controlled conditions; multi-site, multi-year field validation is still lacking.

### 3.2. Pest and Disease Management: Reducing Labor

Accurate and timely pest and disease management is critical for protecting tea yield and quality, which can cause 20–50% yield losses if not controlled early, yet traditional visual scouting is inefficient, labor-intensive, and often subjective [81]. Unlike row crops, tea is a perennial evergreen shrub grown on terraced, often cloudy hillsides, where variable lighting, overlapping leaves, and mixed canopy ages create severe challenges for manual inspection [47,82]. CV offers a potential solution to this challenge by offering a comprehensive solution from automated identification to severity assessment, thereby reducing the labor burden of field scouting and partially replacing manual inspection [48].

At the laboratory proof-of-concept level, early research focused on qualitative identification under controlled conditions; for example, CNNs have achieved high accuracy in classifying images of detached diseased tea leaves [83]. However, deploying such models in complex, variable field environments remains challenging due to the similarity between early-stage lesions and natural leaf spots, as well as occlusion by dense foliage.

At the full-field deployment level, subsequent efforts have shifted towards real-time, in-field detection and localization using robust algorithms like the YOLO series, which balance speed and accuracy. Improved versions have demonstrated detection accuracy exceeding 97% for tea pests in natural settings, meeting practical demands for field monitoring [70]. Thus, CV has proven effective in reducing the need for manual pest scouting, though full replacement of human expertise remains limited by environmental variability.

Moving beyond detection, quantitative assessment of infestation severity is essential for determining economic thresholds and timing interventions [47]. Unlike annual crops where uniform canopies simplify severity estimation, tea plants exhibit heterogeneous shoot growth, requiring pixel-wise or instance-level assessment [82]. Advanced approaches integrate multiple features such as lesion area, color, and texture within fusion strategies or employ end-to-end regression CNNs to estimate severity with minimal labeled data [84]. Furthermore, pest outbreaks in tea are often exacerbated by excessive nitrogen fertilization or water stress, meaning that the same CV-based monitoring system can inform targeted fertilizer and irrigation adjustments, linking pest management with precision nutrient strategies, a direct response to the question of how CV enables more efficient fertilization [85].

Collectively, these technological advances are establishing an intelligent management system capable of early warning, precise identification, and quantitative evaluation, significantly enhancing the efficiency and precision of tea plant protection while reducing unnecessary labor and chemical inputs.

Despite these advances, the majority of pest detection studies have been validated on only one or two tea varieties, and generalizability to other regions and growing conditions remains untested.

### 3.3. Yield Prediction: Supporting Harvest Timing Decisions and Growth Tracking

Accurate yield prediction is vital for tea plantation management and harvest planning [35]. Unlike annual crops, tea is a perennial evergreen producing multiple flushes per season, and yield depends on the proportion of tender buds [86]. Traditional manual shoot counting is laborious and spatially sparse. CV directly addresses this challenge by enabling UAV-based remote sensing for large-scale, high-resolution monitoring. Wang et al. built a UAV tea bud image dataset of 5899 images with 29,958 labeled buds; using YOLOv5 with CSPDarknet53, they achieved a mean average precision (mAP) of 85.63% and estimated spring pre-harvest fresh tea yield at 1223.22 kg/ha [35]. This growth tracking capability illustrates how CV can be applied for growth tracking in the tea industry; farmers can monitor shoot emergence and development over time without manual field visits.

To improve robustness, hybrid frameworks combine remote sensing with crop models or advanced feature selection. Batool et al. compared the AquaCrop simulation model with ten ML regressors; XGBoost achieved the lowest RMSE of 0.154 t/ha using less data, outperforming the process-based model [71]. Jui et al. developed a spatiotemporal hybrid random forest with dragonfly optimization and Support Vector Regression (SVR)-based feature selection, reducing relative prediction error to 11% across 20 tea stations over 40 years [87].

Collectively, these tea-specific studies demonstrate that CV can provide actionable yield forecasts and harvest timing recommendations, thereby supporting both pre-harvest planning and quality preservation through timely picking.

While promising, these yield prediction models have rarely been tested across multiple seasons or farms; their robustness under real-world variability requires further investigation.

### 3.4. Intelligent Harvesting: Reducing Labor and Enabling Selective Plucking

Tea harvesting is uniquely challenging: only tender buds with one leaf are plucked for high-grade teas, the same bush produces multiple flushes per season, and buds must be handled gently to avoid bruising [88]. Addressing the critical shortage and rising cost of skilled harvest labor necessitates autonomous robotic pickers [89]. CV contributes to addressing this challenge by enabling reliable bud detection and picking point localization [90]. The core technological challenge lies in reliable bud detection and picking point localization under variable lighting, occlusion, and dense foliage [90].

For tea bud recognition, deep learning has become the standard under controlled or laboratory conditions. Yang et al. used an improved YOLO-V3 with residual blocks and K-means anchor clustering, achieving over 90% detection accuracy on a high-quality bud dataset [72]. Wang et al. applied Mask-RCNN with ResNet50-FPN, reaching 93.95% accuracy and 92.48% recall on 100 field images [73]. These results, obtained under controlled imaging conditions with standardized lighting and backgrounds, confirm that CV can identify harvestable buds with high precision when environmental factors are favorable. Moving beyond controlled settings, Li et al. combined YOLO with RGB-D imaging, reporting 93.1% detection accuracy and 89.3% recall, with an average localization time of 24 ms per target [34]. These results confirm that CV can identify harvestable buds with high accuracy, directly enabling selective plucking of only the tender shoots.

At the pilot-line level, Yan et al. developed a lightweight MC-DM (DeepLabV3+ with MobileNetV2), reducing model parameters by 89.19% and achieving 16.05 fps inference speed, with picking point recognition accuracies of 82.52% (single bud), 90.07% (bud+ one leaf), and 84.78% (bud+ two leaves) [55]. This represents an intermediate step toward practical deployment, where model compression enables operation on resource-constrained robotic platforms.

These tea-specific studies collectively demonstrate that CV can significantly reduce manual plucking labor, partially replacing human pickers, though occlusion handling and hardware cost remain barriers for smallholder adoption.

It is important to recognize that the reported picking success rates were achieved under relatively favorable conditions; occlusion and lighting variations in dense canopies remain major challenges.

### 3.5. Tea Processing: Detecting Polyphenols, Predicting Flavor, and Preserving Quality

Tea processing, including withering, fermentation, and drying, determines final quality and flavor [91]. Traditional reliance on subjective expertise limits standardization [92]. CV and NIRS can help address this limitation by enabling non-destructive, online monitoring of key parameters, thereby supporting quality preservation decisions during processing [93].

For withering and drying, moisture control is critical. In a pilot-line study conducted under simulated production conditions, Shen et al. combined micro-NIRS with a smartphone and an Elman neural network (ENN) integrated with Principal Component Analysis (PCA), achieving a prediction correlation coefficient of 0.993 for moisture content in black tea withering [74]. At the laboratory level, for sun-drying of Pu-erh tea, Chen et al. used a CNN-Gated Recurrent Unit (GRU) model with environmental parameters (humidity, temperature, radiation) selected by neighborhood component analysis, obtaining moisture prediction RMSE as low as 0.2669 and R^2^ = 0.9882, with RPD values up to 53.6 [94]. These results confirm that CV can support real-time quality preservation during processing by providing accurate moisture control.

Monitoring fermentation is more complex due to biochemical transformations. Critically, CV combined with other sensing technologies can detect polyphenol concentrations and help predict flavor profiles. Several laboratory-level studies have demonstrated high predictive accuracy on controlled sample collections, such as that of Li et al., who applied a laboratory-built CV system and micro-NIRS to Pu-erh pile fermentation; CVS-based texture and color features predicted fermentation degree with 99.30% accuracy, outperforming NIRS alone, and quantified total catechins with RPD = 4.76 [95]. Kimutai et al. developed a CNN model (Tea Net) that outperformed traditional ML (Random Forest (RF), SVM, K-Nearest Neighbors (KNN)) for classifying fermentation stage, achieving >96% accuracy on an image dataset [96]. Huang et al. combined hyperspectral imaging with Particle Swarm Optimization (PSO)-optimized CNN-LSTM, reaching 96.78% test accuracy for black tea fermentation quality [97].

These specific studies demonstrate that CV and data fusion can replace subjective sensory evaluation, enable real-time endpoint determination and consistent quality, and also provide quantitative predictions of catechin content that correlate with flavor characteristics.

Most processing monitoring studies were conducted at laboratories or pilot scale; online integration into production lines is still immature and requires real-time hardware solutions.

### 3.6. Quality Evaluation: The Role of CV in Grading, Authentication, and Shelf-Life Prediction

The final commercial grading of tea directly determines its market value [98]. CV can play an important role in tea classification and scoring by providing objective, repeatable assessment that replaces subjective human sensory evaluation [43]. A multi-technology collaborative approach is central to this advancement. RGB imaging captures appearance traits (color, shape, texture), while HSI provides non-destructive spectral data on internal chemical composition (tea polyphenols, free amino acids) [99]. However, quality evaluation encompasses several distinct prediction tasks, which should be treated separately [76].

For appearance grading, models classify dry tea into commercial grades using expert-labeled visual features; Song et al. developed a segmentation-based method for black tea appearance, using sensory panel scores as ground truth, with random forest achieving R^2^ = 0.898 and RPD = 3.207 [100]. For chemical composition prediction, HSI combined with ML predicts tea polyphenols and free amino acids against HPLC-measured values; Hu et al. reported a prediction coefficient of determination (R^2^_p_) = 0.9248 for polyphenols and 0.8736 for amino acids and achieved 100% accuracy for grade discrimination using SVM [75,76]. For origin authentication and cultivar classification, ResNet50 achieved over 93% accuracy for oolong tea varieties based on labeled cultivar data [101]. For shelf-life prediction, electronic tongue fused with ML reached >98% accuracy for storage time classification using known storage periods as ground truth [102]. Algorithmically, the field has evolved from manually engineered features (color histograms, texture) combined with SVM or Artificial Neural Network (ANN) [103] to deep learning models such as CNNs and hybrid sensor fusion.

These specific studies confirm that CV (often combined with NIRS or electronic sensors) can address multiple quality-related tasks, but each requires task-specific ground truth and evaluation protocols.

Although CV achieves high accuracy in grading and authentication, the reference methods are themselves time-consuming; the cost–benefit trade-off for industrial adoption needs further assessment.

## 4. Challenges in Technology Translation and Industrial Adoption

Despite the significant potential demonstrated by CV technologies across the tea production chain—and notable successes in laboratory and pilot projects—their transition from technical prototypes to standardized industrial solutions faces a series of structural, technical, and economic hurdles. These challenges are inherent to the complexity of the tea industry and are compounded by the current state of AI technology development. This section systematically analyzes the core obstacles to industrial adoption from four perspectives: data, models, system integration, and industrial translation.

### 4.1. The Scarcity of High-Quality, Generalizable Datasets

Data is the critical foundation for AI models, yet acquiring sufficient, high-quality, and representative visual data within the tea industry remains a primary challenge [104]. Tea growth, pest/disease occurrence, and processing dynamics exhibit strong seasonality and regional specificity. Building comprehensive datasets covering diverse seasons, origins, cultivars, and weather conditions requires extensive, long-term monitoring at prohibitive costs [105]. Furthermore, high-quality annotation is labor-intensive, subjective, and relies heavily on domain experts for tasks such as grading disease severity or judging processing states.

Data heterogeneity presents another major barrier. Information is collected from diverse sources, including field RGB cameras, UAV multispectral sensors, handheld NIR devices, and online HSI systems. These generate data with significant differences in format, resolution, scale, and informational dimension. Effectively integrating these heterogeneous, multimodal data streams (topography, weather, imagery) into a unified framework for holistic decision-making remains an unsolved problem [106,107]. Consequently, models trained on limited, localized datasets often suffer from weak generalization to other regions or environmental conditions, creating a key bottleneck for widespread deployment [108,109]. The consequence of such data constraints is now measurable. Wu et al. [110] reported that when a tea shoot detection model trained on a single cultivar was tested on multiple unfamiliar varieties, the AP50 dropped by up to 33.31% compared to its performance on the training variety. A similar pattern appears across broader agricultural vision tasks: plant disease detection models achieving 95–99% accuracy under controlled laboratory conditions typically decline to 70–85% when deployed in open-field scenarios [63]. These figures confirm that cultivar-specific and environment-specific overfitting is not a marginal caveat but a primary barrier to practical deployment. Without explicit cross-validation across diverse cultivars, regions, and seasons, reported accuracy metrics remain poor proxies for real-world reliability.

### 4.2. The Model Trade-Off: Robustness, Real-Time Performance, and Accuracy

Developing vision models robust enough for unstructured field and factory environments is a significant hurdle. Performance can be severely degraded by variable lighting, cluttered backgrounds, target occlusion, and adverse weather. Achieving the necessary robustness requires algorithmic innovations incorporating stronger prior knowledge or advanced techniques like data augmentation and domain adaptation [111].

A fundamental trilemma exists between accuracy, speed, and cost. High-accuracy, complex models typically demand substantial computational resources, hindering real-time inference on edge devices in the field [112]. While lightweight models (MobileNet, tiny YOLO variants) meet real-time requirements for deployment on drones or robots, they often compromise accuracy and stability in complex scenarios [113]. Furthermore, high-fidelity sensors like HSI cameras and LiDAR, though information-rich, carry high costs that limit adoption among small-to-medium enterprises [114]. Balancing precision, latency, and expense under constrained computational budgets is therefore a central engineering challenge.

### 4.3. System Integration and Engineering Hurdles

Integrating standalone vision modules into stable, reliable, and usable industrial systems presents even greater difficulties. Current research often targets isolated tasks, resulting in incompatible systems with disparate data formats and communication protocols. This fragmentation prevents the formation of a seamless, production-wide dataflow and decision-making loop [107].

Hardware limitations are equally critical. Laboratory-grade equipment is frequently ill-suited to harsh field conditions involving dust, humidity, vibration, and temperature extremes. UAVs face trade-offs between flight time, payload capacity, and cost, complicating routine large-scale use. Harvesting robots struggle with maintaining positioning accuracy under vibration and navigating steep, terraced landscapes [106]. For processing, sensors capable of non-destructive internal quality assessment often lack the portability and online capability needed for integration into real-time control loops [115]. Finally, deploying real-time edge computing devices requires careful balancing of computational power, energy consumption, and cost, while cloud–edge collaboration schemes can be hampered by poor network infrastructure in rural areas [116].

### 4.4. Barriers to Industrial Translation and Ecosystem Development

Successful technology adoption hinges on broader economic and social factors. A critical barrier is the acute shortage of interdisciplinary talent possessing deep expertise in both tea science (biology, agronomy, processing) and AI/engineering. This gap can lead to solutions that are technically sound but misaligned with practical production needs [117].

Economic viability is paramount for end-users. For many tea farmers and small enterprises, the initial investment, ongoing maintenance, and operational learning curves associated with CV systems are prohibitive. A clear and compelling return on investment (ROI) coupled with user-friendly operation is essential for adoption, yet many current solutions are too costly and complex. Quantitatively, the lab-to-field performance gap is non-negligible. While tea bud detection models routinely report >93% accuracy on curated datasets, field trials using identical algorithms yield picking success rates of approximately 83%, a ~10 percentage-point decline primarily attributable to illumination changes, occlusion, and mechanical vibration [34,72]. In plant disease detection more broadly, accuracy typically drops by 15–20% when transitioning from controlled chambers to open fields [63]. These figures underscore that accuracy reported in isolation is an insufficient proxy for deployability; without explicit reporting of field performance degradation, practitioners cannot reliably benchmark candidate solutions.

Finally, the diversity of tea cultivars and processing techniques resists a one-size-fits-all solution. Widespread scaling requires extensive localization and tuning. The absence of industry-wide technical standards, data protocols, and evaluation benchmarks further increases implementation complexity and hinders replication [7,118].

To address these interconnected challenges, a conceptual framework is proposed to guide the transition of CV technologies from laboratory research to field-deployable industrial solutions. This framework operates across four dimensions: (1) establishing shared data ecosystems with standardized protocols to improve model generalizability; (2) developing domain adaptation and few-shot learning techniques to bridge the lab-to-field performance gap; (3) designing modular and interoperable system architectures with edge–cloud hybrid computing for real-time deployment; and (4) reducing economic barriers through low-cost hardware and user-friendly interfaces, alongside cross-disciplinary training to cultivate the necessary talent pool. These dimensions are not independent but mutually reinforcing, and their implementation should be adapted to specific application contexts through collaboration among researchers, engineers, industry stakeholders, and policymakers.

The deepening application of CV in the tea industry is at a critical juncture, transitioning from laboratory validation to field deployment. The successful implementation of the above framework requires concerted efforts beyond algorithmic innovation, encompassing collaborative data ecosystem development, purpose-built hardware engineering, robust system integration, cost optimization, and cross-disciplinary expertise cultivation. Only through such a systematic and collaborative approach can the transformative potential of this technology for the tea industry be fully realized.

## 5. Conclusions and Future Perspectives

This review has provided a task-oriented analysis of AI vision technologies across the tea production chain, from cultivation to quality evaluation. Based on the identified gaps, future research should focus on several concrete targets.

First, the community should build a public tea benchmark dataset containing at least ten cultivars, three growth stages, multiple lighting conditions, and 50,000 labeled instances. Such a resource would enable fair algorithm comparison and reduce overfitting. Second, domain adaptation and few-shot learning methods should be developed to limit the lab-to-field accuracy drop to less than five percentage points. Third, affordable edge vision systems such as a portable multispectral device costing below $500 and a lightweight CNN model achieving mAP > 0.90 at >30 FPS on a sub−10 W embedded processor are urgently needed. Additionally, every future study should report the TRL, validation conditions, and cost breakdown to enable fair comparison across different approaches. Currently, RGB imaging is the most mature technology (TRL 8–9) and has been widely deployed, whereas HSI and LiDAR remain at lower maturity levels (TRL 4–5) due to high cost and data complexity. NIRS, TIC, and UAV fall in the intermediate range (TRL 6–7), with pilot-scale validation demonstrated but barriers to routine deployment remaining.

Achieving these goals will help transform CV from a laboratory promise toward practical tools for the tea industry, supporting more standardized, efficient, and sustainable production. Looking further ahead, the deep integration of CV holds transformative potential to reshape traditional production modes and quality control systems, provided that continued efforts are made in creating shared data resources, advancing adaptive algorithms, developing affordable and robust hardware, and fostering interoperable system architectures. Only then can CV become a true enabler for the intelligent and high-quality development of the global tea sector.

## Figures and Tables

**Table 1:** Comparison of multidimensional vision technologies in the tea industry.

Category	Technology	Core Benefits	Key Applications in Tea Industry	Example Performance	Validation Setting	Cost	TRL 1–9	Ref
Imaging modality	RGB imaging	Low cost, easy deployment, mature technology	Bud maturity detection, disease screening, dry tea grading	Accuracy: 96.8%	Field, 2000+ images	low	8–9	[16,17]
Imaging modality	HSI	Spatial+ spectral data; non-destructive internal analysis	Moisture/chemical mapping, internal quality assessment, early stress diagnosis	R^2^ > 0.91	Lab, 100–200 samples	High	4–5	[18,19]
Spectroscopic technique	NIRS	Rapid, portable, real-time, non-destructive and integrable into production lines	Moisture monitoring, fermentation degree, key component prediction	R^2^ = 0.976	Pilot line or lab, 300+ batches	Medium	6–7	[20]
Imaging modality	TIC	Non-contact temperature mapping; detects stress & anomalies	Water stress assessment, uneven heating detection, mold growth indication	R^2^ = 0.84, 0.77 and 0.79	Field, 50+ plots	Medium-High	6–7	[21]
3D spatial sensing	LiDAR	Accurate 3D depth info; robust to lighting/texture	Tea bud 3D localization, canopy analysis, biomass estimation	R^2^ = 0.75–0.84	Field, 500+ buds	High	4–5	[22]
Aerial platform	UAV	Large-scale, terrain-agnostic, multi-temporal monitoring	Field vigor mapping, pest/disease foci, yield prediction	R^2^ = 0.97	Field, multi-temporal, 50+ ha	Medium-High	6–7	[23,24]

**Table 2:** Algorithm selection and comparative analysis for CV tasks in the tea industry.

Scenario/Task Type	Recommended Algorithms	Key Advantages	Applicable Conditions	Main Limitations	Hardware Requirements	Ref
Data-limited, near-linear problems	SVM, PLSR	Simple, low computational cost, strong interpretability	Small sample size, approximately linear feature relationships	Poor performance on highly nonlinear problems, complex backgrounds, or variable lighting	Low	[49]
Unstructured field/plant challenges	CNN	Automatically learns robust features; adapts to complex environments	Sufficient labeled data available	Requires large, annotated datasets; limited interpretability	Moderate	[48]
Fast image-level classification	Lightweight CNN (MobileNet)	Balances speed and accuracy; suitable for edge deployment	High real-time demands, constrained computational resources	Slightly lower accuracy than full-sized models	Low to moderate	[52]
Object detection & localization	YOLO series	End-to-end, efficient inference, good real-time performance	High frame-rate requirements, mobile platforms	Higher miss rate for small targets (early-stage lesions, tender buds)	Moderate	[53]
Pixel-wise fine segmentation	FCN, Mask R-CNN	Pixel-level precision; instance segmentation capable	Requires accurate contours or region boundaries	High computational cost; typically needs GPU; difficult for real-time edge deployment	High	[54]
Pixel-wise fine segmentation	DeepLab variants	High accuracy for irregular targets (disease spots, tea buds)	When instance-level detail is required; sufficient labeled data available	Higher computational cost than lightweight CNNs; requires GPU	High	[55]
Long-range spatial dependence	ViT	Captures global context in canopy or processing images	Large-scale classification with abundant data	Data-hungry; not yet validated on small tea datasets	Very high	[56]

**Table 3:** Key environmental differences between laboratory research and field application in tea gardens/processing facilities.

Dimension	Laboratory Environment	Tea Garden/Factory	Specific Impact	Ref.
Lighting conditions	Constant, uniform, controllable	Highly variable natural light (cloud cover, time of day), artificial light interference	Unstable color features; segmentation/detection accuracy may drop	[62]
Background complexity	Single, uniform background (whiteboard, black cloth)	Weeds, soil, dead leaves, processing equipment, personnel movement	Difficulty separating targets from background; increased false positive rate	[66]
Target pose and occlusion	Fixed viewpoint, intact target, standard pose	Buds occluded by leaves, tilted angles, shaking during harvesting	Key-point localization bias; high miss rate for small targets	[67]
Data acquisition equipment	High-precision industrial cameras, HSI, LiDAR	Consumer-grade RGB cameras, low-cost depth sensors, smartphones	Lower image resolution; lack of depth/spectral information; poor input quality	[63]
Environmental stability	Constant temperature/humidity, vibration-free, dust-free	Temperature fluctuations, vibration, dust, rain/fog	Sensor drift; increased data noise; poor repeatability	[68]

**Table 4:** Overview of computer vision technology applications across the tea production chain.

Application Stage	Setting	Specific Task	Sensing Technology	Modeling Approach	Key Outcome	Ref.
Smart Cultivation	Field/Aerial	Monitoring nitrogen content, chlorophyll, leaf area index, canopy temperature, water stress.	HSI, TIC, RGB	CNN, basic ML algorithms	Enables precise, quantitative irrigation/fertilization; increases yield/quality while reducing environmental impact.	[69]
Pest & Disease Control	Field	Automated pest/disease identification, localization, and severity assessment.	RGB	YOLO series, CNN, Multi-feature fusion	Facilitates early warning, precise identification, and quantitative evaluation for targeted intervention.	[70]
Yield Prediction	Aerial/Satellite	Regional yield estimation, harvest zone identification.	UAV	Random Forest, DL models, Hybrid mechanistic-data models	Enhances the efficiency and scientific basis of crop protection and harvest planning.	[35,71]
Intelligent Harvesting	Field	Tea bud recognition, precise picking point localization.	RGB, LiDAR	YOLO series, Mask R-CNN, Lightweight network architectures	Addresses labor shortages; enables autonomous picking with high success rates.	[72,73]
Processing	Processing Facility	Moisture prediction during withering/fixation, fermentation monitoring, drying endpoint determination.	NIRS, HSI, CV	SVM, CNN, CNN-LSTM hybrids	Enables online, non-destructive, and dynamic monitoring of critical processes, promoting standardization.	[74]
Quality Evaluation	Lab/Production Line	Appearance grading, chemical composition prediction, origin authentication, shelf-life prediction	HSI, CV	Multivariate statistical analysis, ML/DL models	Establishes an objective, efficient, and integrated system for quality assessment and grading.	[75,76]

## Data Availability

No new data were created or analyzed in this study. Data sharing is not applicable to this article.
